# Antibiotic consumption in hospitals in humanitarian settings in Afghanistan, Bangladesh, Democratic Republic of Congo, Ethiopia and South Sudan

**DOI:** 10.1186/s13756-024-01449-7

**Published:** 2024-08-15

**Authors:** Kristina Skender, Gabriel Versace, Annick Danyele Lenglet, Kate Clezy

**Affiliations:** 1 Médecins Sans Frontières, Operational Centre Amsterdam (OCA), Amsterdam, The Netherlands; 2 Department of Global Public Health, Health Systems and Policy, Karolinska Institutet, Stockholm, Sweden; 3 International Centre for Antimicrobial Resistance Solutions (ICARS), Copenhagen, Denmark; 4 Antimicrobial Research Unit, School of Health Sciences, University of KwaZulu-Natal, Durban, South Africa; 5 Clinical Excellence Commission New South Wales, Sydney, Australia

**Keywords:** Antibiotic consumption, Antimicrobial resistance, Hospital, Humanitarian, Afghanistan, Bangladesh, Democratic Republic of Congo, Ethiopia, South Sudan

## Abstract

**Background:**

Antimicrobial resistance is of great global public health concern. In order to address the paucity of antibiotic consumption data and antimicrobial resistance surveillance systems in hospitals in humanitarian settings, we estimated antibiotic consumption in six hospitals with the aim of developing recommendations for improvements in antimicrobial stewardship programs.

**Methods:**

Six hospitals supported by Médecins sans Frontières were included in the study: Boost-Afghanistan, Kutupalong-Bangladesh, Baraka and Mweso-Democratic Republic of Congo, Kule-Ethiopia, and Bentiu-South Sudan. Data for 36,984 inpatients and antibiotic consumption data were collected from 2018 to 2020. Antibiotics were categorized per World Health Organization Access Watch Reserve classification. Total antibiotic consumption was measured by Defined Daily Doses (DDDs)/1000 bed-days.

**Results:**

Average antibiotic consumption in all hospitals was 2745 DDDs/1000 bed-days. Boost hospital had the highest antibiotic consumption (4157 DDDs/1000 bed-days) and Bentiu the lowest (1598 DDDs/1000 bed-days). In all hospitals, Access antibiotics were mostly used (69.7%), followed by Watch antibiotics (30.1%). The most consumed antibiotics were amoxicillin (23.5%), amoxicillin and clavulanic acid (14%), and metronidazole (13.2%). Across all projects, mean annual antibiotic consumption reduced by 22.3% during the study period, mainly driven by the reduction in Boost hospital in Afghanistan.

**Conclusions:**

This was the first study to assess antibiotic consumption by DDD metric in hospitals in humanitarian settings. Antibiotic consumption in project hospitals was higher than those reported from non-humanitarian settings. Routine systematic antibiotic consumption monitoring systems should be implemented in hospitals, accompanied by prescribing audits and point-prevalence surveys, to inform about the volume and appropriateness of antibiotic use and to support antimicrobial stewardship efforts in humanitarian settings.

**Supplementary Information:**

The online version contains supplementary material available at 10.1186/s13756-024-01449-7.

## Introduction

Antimicrobial resistance (AMR) represents a critical global public health threat with predictions of increased morbidity and large numbers of premature deaths [1–3]. Despite limitations in data quality and availability, there is consensus that the AMR burden is greatest in low- and middle- income countries (LMICs) [4–6]. Populations in LMICs already experience a disproportionately high burden of communicable diseases and the consequences of antimicrobial resistant infections may be catastrophic at an individual, household and community level, due to increased morbidity, mortality, healthcare costs and loss in the work ability [3, 6, 7]. The evolution and spread of AMR in LMICs may be further hastened by the lack of universal healthcare including vaccination; inadequate water, sanitation and hygiene systems; inequities in access to medicines; unregulated sales of antibiotics; limited diagnostic capability; lack of prescribing guidelines and surveillance; lack of public awareness and education; and the presence of weak healthcare systems [8, 9]. Weak healthcare systems are those that face inadequate infrastructure, shortage of workforce, limited access to medicines and technology, lack of health financing, poor governance, inefficient health information systems, inadequate service delivery, and low community engagement and trust [10]. Many of these challenges experienced in LMICs are similar and exacerbated in humanitarian settings.

Rising antibiotic consumption is one of the main drivers of the increasing rates of AMR [5, 11, 12]. To support local and national antibiotic stewardship plans, in 2015, the WHO developed Global Action Plan on AMR [13], based on which the majority of countries developed their national action plans on AMR. However, only 20% of countries with national action plans are actively monitoring their implementation [14]. Furthermore, in order to prevent inappropriate use of antibiotics, the World Health Organization (WHO) developed Access Watch Reserve (AWaRe) classification, by which antibiotics are grouped into three categories based on their preference for use and potential for developing resistance [15]. In addition, the WHO has set a target that by 2023, at least 60% of all consumed antibiotics on the national level should be from the Access group. Of particular concern is a global increase in the consumption of Reserve antibiotics, defined by the WHO as antibiotics that should be reserved for the treatment of multi-drug resistant infections [15]. It is estimated that the global consumption of carbapenems and polymyxins increased by 45% and 13%, respectively, between 2000 and 2010 [12]. However, these reports are mostly derived from national surveys of antibiotic sales [12] and cannot be generalized to all settings.

The WHO Global Action Plan on AMR (2015) stated that monitoring of antimicrobial consumption should be an integral part of every healthcare facility antimicrobial stewardship program and used to inform strategies for optimal antimicrobial use [13]. Reproducible indicators for antimicrobial consumption require information about dispensing as well as a measure of patient activity, often expressed as patient-days, or patient bed-days [16]. Other methods have also been suggested towards improved antimicrobial stewardship, including antibiotic prescribing surveys, closed-loop audits, quality improvement and educational programs. These interventions have been associated with a reduction in the incidence of healthcare-associated infections, patient morbidity and mortality, and improvement in patient safety [17–19].

A lack of antibiotic consumption data and robust surveillance systems to monitor AMR in LMICs hinders our understanding of the AMR burden, particularly in Africa, South-East Asia, and the Middle East [12, 13]. A recent meta-analysis of 52 studies reviewed the association between antibiotic consumption and antimicrobial stewardship programs, but only four studies from LMICs were included in the meta-analysis; three from China and one from Iran [20]. Within LMICs, the majority of antibiotic consumption and bacterial resistance data is from tertiary-care centers in urban settings [21, 22]. In healthcare facilities in humanitarian settings, a structured system for antibiotic consumption monitoring is largely missing and not considered a priority [7, 23]. Additional challenges commonly encountered in humanitarian settings that negatively impact antibiotic use are: unavailability and inaccessibility of healthcare facilities, excessive workload of medical staff, disruption or complete absence of medical supply, and substandard quality of medicines in the market [7, 24–27].

Médecins sans Frontières (MSF) is an international independent medical humanitarian organization that has been providing medical assistance since 1971, in settings of natural disasters, armed conflicts and civil unrests, epidemics, internal and external displacement, and exclusion from healthcare [28]. MSF recognizes that in the provision of healthcare there is a responsibility to work towards improving the use of antimicrobials, both to ensure access and appropriate use of antimicrobials. Addressing AMR within MSF supported health facilities covers three pillars: (i) infection prevention and control, (ii) antimicrobial stewardship and (iii) microbiology and surveillance [26, 29]. Implementation of each of the pillars is challenging in resource-limited and unstable settings [26]. Despite this overall AMR strategy, there is currently no structured approach in MSF supported healthcare facilities to monitor antibiotic consumption. Nevertheless, MSF recognizes the importance of having accurate estimates of antibiotic consumption within health structures to inform ongoing antibiotic stewardship activities. Therefore, the aim of this study was to estimate antibiotic consumption in six MSF supported hospitals in order to develop recommendations for improvements in antimicrobial stewardship programs.

## Materials and methods

### Study settings

Six MSF supported hospitals were included in the study: Boost-Afghanistan, Kutupalong-Bangladesh, Baraka and Mweso-Democratic Republic of Congo (DRC), Kule-Ethiopia, and Bentiu-South Sudan (Table 1). Hospitals in Kutupalong, Kule and Bentiu were fully managed by MSF, whereas hospitals in Boost, Baraka and Mweso were Ministries of Health hospitals supported by MSF during the time of the study. These hospitals provide a good representation of a variety of secondary healthcare facilities supported by MSF globally, including two refugee camps (Kutupalong and Kule), one internally displaced person camp (IDP; Bentiu) and three hospitals in the context of chronic conflict (Boost, Mweso and Baraka). The latter hospitals all had over 100 beds covering similar medical services including internal medicine, maternity, intensive care unit (ICU), and surgery. The hospitals in refugee and IDP camps had less beds and covered fewer medical activities (Table 1). Disease burden and highest priority medical needs differ significantly between each of these hospitals.

**Table 1:** MSF project hospitals and their set up

Country	Hospital	Humanitarian context	Number of beds	Wards
Afghanistan	Boost	Chronic conflict	109	Maternity
				Internal medicine
				ICU
				Isolation
				Surgery
Bangladesh	Kutupalong	Refugee camp	36	Internal medicine
				Isolation
DRC	Baraka	Chronic conflict	108	Internal medicine
				ICU
				Maternity
				Surgery
	Mweso	Chronic conflict	147	Internal medicine
				ICU
				Maternity
				Surgery
Ethiopia	Kule	Refugee camp	62	Internal medicine
				High dependency unit
				Maternity
South Sudan	Bentiu	IDP camp	60	Internal medicine
				Maternity
				Surgery

### Data collection

This was a retrospective study of secondary collected data. Inpatient data was collected from 2018 to 2020 for all inpatients from two MSF data collection tools: (1) District Health Information System (DHIS) Excel tool and online system (DHIS2), and (2) pharmacy Consumption Tool (CT). The DHIS2 collected individual inpatient information on age and dates of admission and discharge. The CT provided the dispensing data of all antibiotics in each hospital, from the central hospital pharmacy to the relevant medical wards.

### Data analysis

From the CT, we grouped antibiotics based on the WHO Anatomical Therapeutic Chemical (ATC) classification [30] and the WHO Access Watch Reserve (AWaRe) classification [15]. We calculated bed-days for all adult admissions (> 15 years of age) as per the WHO guidance [16] using patient admission and discharge data from DHIS2. Antibiotic use was calculated by the metric Defined Daily Doses per 1000 bed-days (DDDs/1000 bed-days) for every hospital, using formula *DDD of antibiotic = consumption in units x grams per unit / WHO DDD for the antibiotic*. As DDD values are only available for adults [30], pediatric patients below 15 years of age were excluded from the analysis. Antibiotics dispensed to the pediatric or neonatal wards, as well as pediatric formulations such as oral suspensions were excluded from the analysis. Mean values of DDDs/1000 bed-days were compared between the project hospitals. Statistical analysis was performed using R version 4.0.3.

## Results

In total, 36,984 inpatients above the age of 15 from six project hospitals from 2018 to 2020 were included in the study (Table 2.) The mean age did not vary significantly between hospitals (p-value = 0.97), but patients in Boost-Afghanistan were the eldest and those admitted in Baraka-DRC were the youngest. The longest mean length of stay was recorded in Bentiu-South Sudan (5.2 days) and the shortest in Kutupalong-Bangladesh (2.6 days) (Table 2).

**Table 2:** Inpatients’ characteristics in six MSF project hospitals between 2018 and 2020

Hospital	Patients, *N*	Age, years	Length of stay, days
		Mean (SD)	Mean (SD)
Boost	9595	37 (17)	3.0 (3.2)
Kutupalong	9245	30 (15)	2.6 (3.0)
Baraka	1901	28 (9)	3.3 (4.1)
Mweso	8045	30 (12)	4.0 (5.0)
Kule	2398	33 (15)	4.6 (6.2)
Bentiu	5800	34 (16)	5.2 (7.5)

**Fig. 1:**
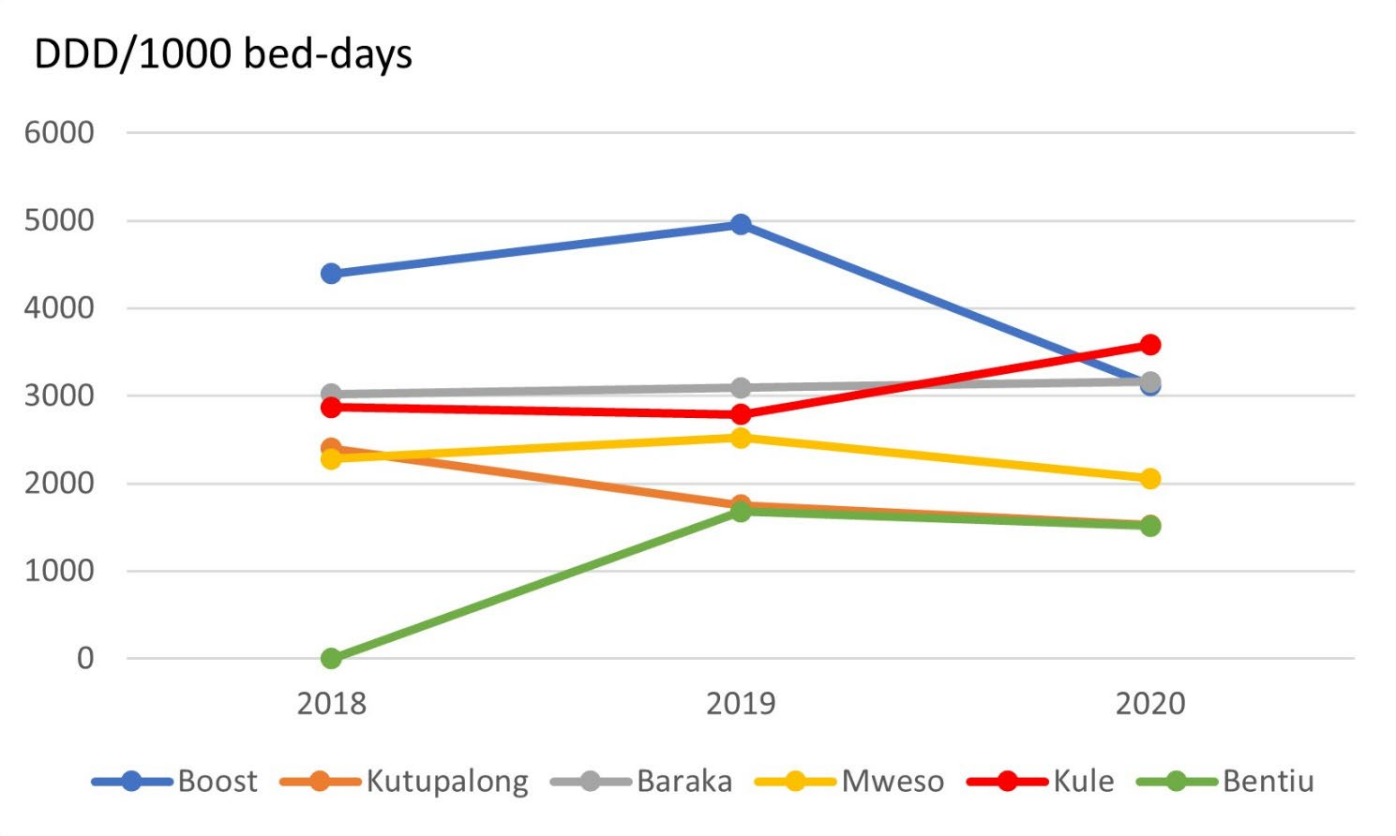
Total antibiotic consumption by DDDs/1000 bed-days in six MSF project hospitals between 2018 and 2020

The total antibiotic consumption by hospital over the study period is shown in Fig. 1. Antibiotic consumption varied between project hospitals with Boost-Afghanistan showing the highest DDDs/1000 bed-days (4157) for the study period, and Bentiu-South Sudan the lowest (1598). The average antibiotic consumption in all hospitals was 2745 DDDs/1000 bed-days. Across all project hospitals, the mean annual antibiotic consumption reduced from 3212 DDDs/1000 bed-days in 2018 to 2494 DDDs/1000 bed-days in 2020, which is a reduction of 22.3%.

**Fig. 2:**
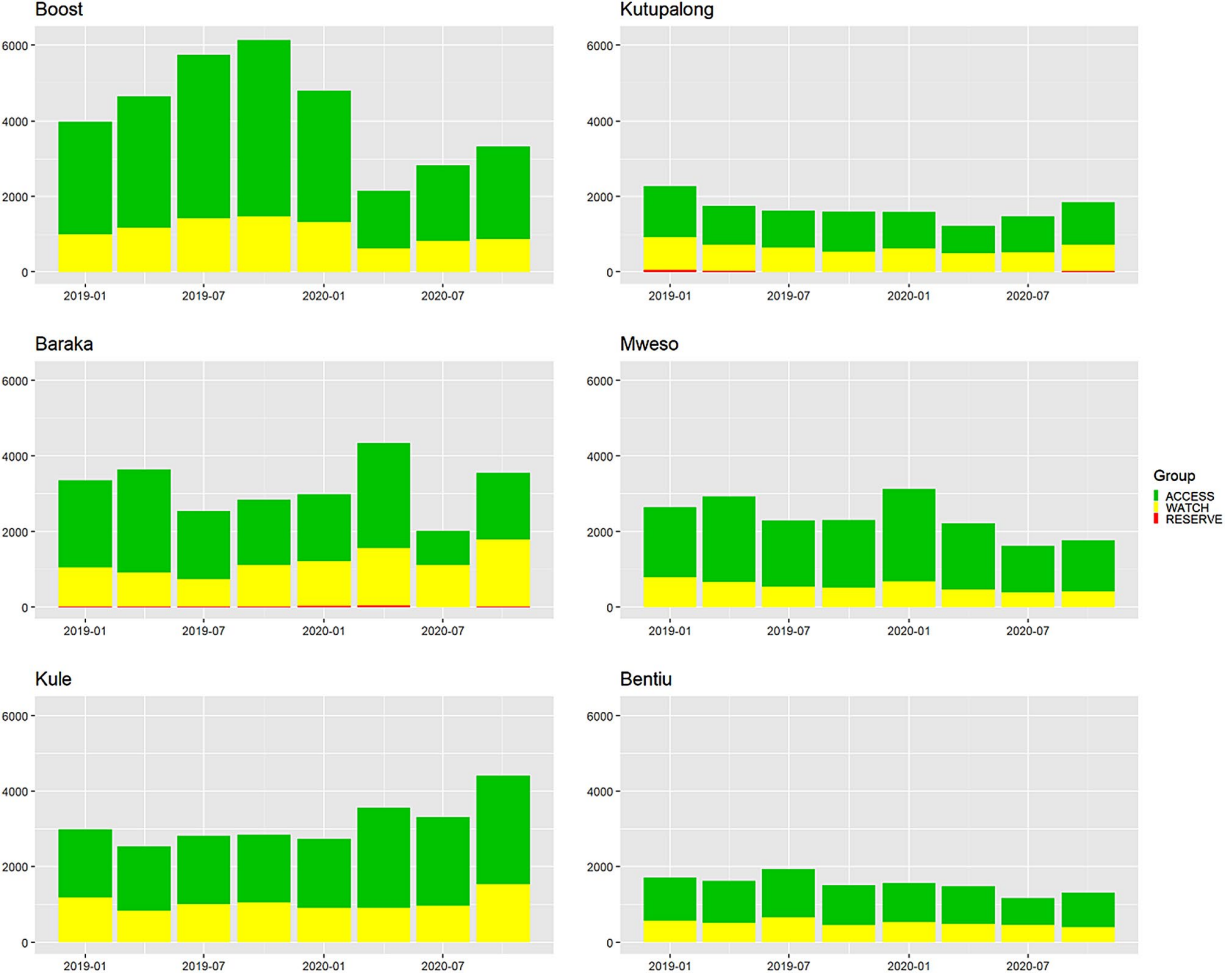
Consumption of AWaRE antibiotics by DDDs/1000 bed-days in six MSF project hospitals between 2018 and 2020

Each hospital had different patterns of antibiotic usage. The most consumed antibiotics in all hospitals were from the Access group (69.7%), followed by the Watch group (30.2%), with the remaining 0.1% being from the Reserve group (Table 3). The proportion of Access antibiotics was the highest in Boost (73.9%) and lowest in Bentiu (58.8%). Subsequently, the proportion of Watch antibiotics was highest in Bentiu (41.2%) and lowest in Boost (26.1%). Reserve antibiotics were only used in Kutupalong (1.1%) and Baraka (0.4%), while other hospitals did not use them during the study period (Table 3.). In all six hospitals, the most consumed antibiotics were: amoxicillin (23.5%), followed by amoxicillin and clavulanic acid (Amoxiclav) (14%) and metronidazole (13.2%) (Table 4.). Some Watch antibiotics were among the top three most consumed antibiotics: ceftriaxone in Kutupalong, Bentiu, Kule and Mweso, and cefixime in Baraka hospital (Table 4). The total antibiotic consumption per AWaRe classification expressed by DDDs/1000 bed-days is given in Fig. 2.

**Table 3:** Consumption of antibiotics per AWaRe classification in six MSF project hospitals between 2018 and 2020

MSF project hospital							
Group	Boost	Kutupalong	Baraka	Mweso	Kule	Bentiu	Total
Access	73.9%	60.4%	64.8%	73.75%	64.4%	58.8%	69.7%
Watch	26.1%	38.5%	34.8%	26.25%	35.6%	41.2%	30.2%
Reserve	0%	1.1%	0.4%	0%	0%	0%	0.1%

**Table 4:** Annual consumption of most consumed antibiotics in six MSF project hospitals between 2018 and 2020

Hospital	Antibiotic 1	DDDs/ 1000 bed-days	Antibiotic 2	DDDs/ 1000 bed-days	Antibiotic 3	DDDs/ 1000 bed-days
		Mean (SD)		Mean (SD)		Mean (SD)
Boost	Amoxiclav*	1114 (132)	Amoxicillin	663 (292)	Metronidazole	579 (292)
Kutupalong	Cloxacillin	368 (70)	Ceftriaxone	265 (97)	Metronidazole	234 (50)
Baraka	Amoxicillin	1141 (254)	Cefixime	473 (137)	Metronidazole	405 (41)
Mweso	Amoxicillin	1034 (172)	Metronidazole	405 (57)	Ceftriaxone	176 (35)
Kule	Amoxicillin	728 (117)	Cloxacillin	451 (71)	Ceftriaxone	417 (50)
Bentiu	Benzylpenicillin	283 (89)	Ceftriaxone	240 (31)	Amoxiclav*	164 (10)

*Amoxiclav = amoxicillin and clavulanic acid

## Discussion

Results of this study show that in the six MSF supported hospitals in humanitarian settings, antibiotic consumption varied substantially between 2018 and 2020 (highest in Boost hospital-Afghanistan and lowest in Kutupalong hospital-Bangladesh). The majority of consumed antibiotics in all project hospitals were from the Access group (amoxicillin, amoxicillin and clavulanic acid, and metronidazole). We also observed that during the study period, antibiotic consumption reduced across all six hospitals by 22.3%, mainly driven by the reduction in Boost hospital. There is very scarce literature on antibiotic consumption in hospitals within humanitarian settings, and our study will start to generate some much-needed evidence on this topic.

We observed a much higher antibiotic use in the hospitals in our study compared to antibiotic use rates reported from other hospital settings. A systematic review of antibiotic consumption in 3130 acute care hospitals worldwide, between 1997 and 2013, found a pooled rate of 586 DDDs/1000 hospital days for all antibiotics [31]. This review found the highest levels of antibiotic consumption in intensive care units and in medium sized, private, or teaching hospitals in Europe. In our study, the average consumption was 4.7 times higher (2745 DDDs/1000 bed-days). However, the majority of included hospitals in this systematic review were from high-income countries [31]. Therefore, it is difficult to compare these numbers with those calculated in our study, as the patient population and the most prevalent diseases that require hospitalization in our hospital settings are likely to be very different from those in Europe.

There is a paucity of studies which measure antibiotic consumption using DDD indicators in other hospitals from the countries included in our study and from other humanitarian settings. In general, antibiotic consumption in our study was higher compared to the consumption in a few published studies from other LMICs. In one hospital in South Africa, with data taken before and after a stewardship intervention, total antibiotic consumption reduced from 1046 DDDs/1000 bed-days in 2011 to 868 DDDs/1000 bed-days in 2013 (reduction of 17%) and remained at similar levels by 2015 [17]. In another study from Ethiopia conducted from 2013 to 2014, mean antibiotic consumption was 810.6 DDD/1000 bed-days, which was again notably lower than in our study. However, this study only measured consumption in three inpatient medical wards [19]. Additionally, the results from one Eritrean study in selected medical wards in two hospitals from 2014 to 2018, showed that total antibiotic consumption was 1585 DDDs/1000 bed-days in the tertiary referral hospital and 1176 DDDs/1000 bed-days in the secondary hospital [18], which again was lower compared to the average consumption in all hospitals from our study. The antibiotics with the highest consumption in the Eritrean study were also somewhat different. In the Eritrean hospitals, the consumption of benzylpenicillin (87.8; 35.4 DDDs/100 bed-days) was higher than in Bentiu hospital-South Sudan (283 DDDs/1000 bed-days), whereas the consumption of amoxicillin (16.9; 4.3 DDDs/100 bed-days) and ceftriaxone (10.1; 6.0 DDDs/100 bed-days) was lower than in all our project hospitals [18].

The majority of consumed antibiotics in all project hospitals belonged to the Access group (69.7%), and therefore the overall selection of antibiotics reached the WHO target of at least 60% of antibiotic consumption from the Access category, which might broadly imply the appropriate selection of antibiotics [15]. However, the consumption of Access antibiotics in Bentiu hospital (58.8%) was slightly below the target of 60%. Nevertheless, this WHO recommendation refers to the overall antibiotic use at national level and not only at hospital level, where more serious clinical infections are treated compared to primary-care facilities [15]. Overall, the most consumed antibiotic was amoxicillin, and the top three consumed antibiotics were mostly Access antibiotics in each hospital. However, Watch antibiotics like ceftriaxone were commonly used in Kutupalong, Mweso, Kule and Bentiu, as was cefixime in Baraka hospital. As ceftriaxone is the most recommended parenteral antibiotic in the MSF antibiotic guidelines for many common infection syndromes requiring hospitalization, relatively high usage is expected. Reserve antibiotics were only seldom used in Kutupalong (1.1%) and Baraka (0.4%), while other project hospitals had no access to antibiotics classified in the Reserve category. We have insufficient information to explain the rates and reasons for the use of Reserve antibiotics in Kutupalong and Baraka hospitals. As there was no access to microbiology, i.e., culture and sensitivity testing, in any project hospital at the time of the study, the use of Reserve antibiotics was not encouraged. Equitable and responsible access, as well as continuous monitoring of the use of Watch and Reserve antibiotics is essential in the development of antimicrobial stewardship programs in humanitarian settings.

Variations in antibiotic consumption between hospital settings can be partially explained by variations in the types of admitted patients (case-mix). For example, Boost hospital in Afghanistan treated a significant number of patients with trauma and had a busy surgical service, whereas Kutupalong hospital in Bangladesh did not have a surgical service, referring all these patients to another facility. Additionally, several countries, including Afghanistan, have noted a difference in the perceived value of antibiotics and expectations from medical assessment, which may drive differences in consumption [27]. Seasonal variation in antibiotic consumption has been shown in many countries [32] to align with either a rainy season or winter. Additionally, in many humanitarian settings there are seasonal differences in rates of infectious diseases and therefore, associated variation in antibiotic consumption is expected.

Total antibiotic use in all project hospitals reduced between 2018 and 2020 and the reasons for this are not completely clear. All study countries, except South Sudan, developed national action plans on AMR [33], although commitment to implementation has been largely missing [14]. There may have been some impact from the MSF antimicrobial stewardship focal points in the hospitals, however due to the lack of information about the case-mix and how it might have changed over time, it is difficult to assess the overall decline in antibiotic consumption. The lack of diagnostic capability combined with high staff turnover, regional insecurity, conflict and procurement instability would contribute to variation in antibiotic consumption over time. COVID-19 might have had some impact in early 2020, as in some hospitals (e.g., Boost-Afghanistan), a separate COVID-19 facility was opened to which patients with suspected COVID-19 (i.e., all respiratory infections) were referred to for further management. This may partially explain the reduction in antibiotic consumption in Boost hospital in the early part of 2020. It has to be noted that the reduction in overall antibiotic use was largely driven by reduction in Boost hospital.

The results of this study suggest that there is a high consumption of antibiotics in hospitals in humanitarian settings relative to other settings. However, in the absence of robust comparative data, and an understanding of the case-mix, it is not possible to know whether these represent unacceptable levels of antibiotic use. Information about appropriate prescribing, which can be obtained by e.g., repeated point prevalence surveys, was unavailable in most hospital projects and would clarify whether the consumption was appropriate for the patient load and complexity. Antibiotic consumption often declines when antimicrobial stewardship programs are introduced, and during the study period there were limited antimicrobial stewardship efforts in two projects: Bentiu-South Sudan and Boost-Afghanistan. All project hospitals had available MSF antibiotic prescribing guidelines. Bentiu hospital performed a point prevalence survey in 2019, the results of which showed relatively good antibiotic guidelines compliance. It must be noted, however, that the MSF antibiotic prescribing guidelines are based on a limited range of antibiotics, with ceftriaxone often as a first choice.

This study had several limitations. Pharmacy data for Bentiu hospital in 2018 was inconsistent and had unusual outliers, and therefore was excluded. Furthermore, we were unable to compare the antibiotic consumption between the project hospitals, considering the variability of the contexts, health infrastructures, services and endemicities. In other words, only a comparison of antibiotic consumption within a single hospital over several years would be reliable. However, assuming that MSF project hospitals had a similar case-mix and medical supply problems each year, the results should be reasonably comparable on a year-on-year basis within a facility. It would have been useful to have more detailed information about changes in the medical services mix (e.g., increasing or ceasing surgical services), or in case-mix (e.g., an influx of patients due to population displacement) to explain the variation in antibiotic consumption. Further investigation into individual infectious diagnoses and corresponding antibiotic treatment over several seasons is required to investigate the potential seasonality of antibiotic consumption patterns. In addition, MSF data collection tools did not provide individual patient dispensing information; therefore, we were unable to estimate other metrics of antibiotic use. Linking pharmacy dispensing data to individual diagnoses would have provided more information about the appropriateness of antibiotic use; however, it was out of the scope of this study.

## Conclusion

This was the first study to assess antibiotic consumption by DDD metric in hospitals in humanitarian settings. The results of this study show that antibiotic consumption was high compared to the other settings; however, in the absence of comparative studies and case-mix information, it is uncertain if the high antibiotic consumption was inappropriate. This study highlights the importance of establishing routine systematic antibiotic consumption monitoring systems in hospitals in humanitarian settings. Such routine systems should be complemented with prescribing audits and point prevalence surveys to assess the appropriateness and further guide healthcare staff in optimal antibiotic use, as well as to support antimicrobial stewardship efforts.

### Electronic supplementary material

Below is the link to the electronic supplementary material.

Supplementary Material 1

## Data Availability

MSF has a managed access system for data sharing. Data are available on request in accordance with MSF’s data sharing policy. Requests for access to data should be made to data.sharing@msf.org. For more information please see: (1) MSF’s Data Sharing Policy: http://fieldresearch.msf.org/msf/handle/10144/306501, (2) MSF’s Data Sharing Policy PLOS Medicine article: http://journals.plos.org/plosmedicine/article?id=10.1371/journal.pmed.1001562.

## Abbreviations

AMR: Antimicrobial resistance
ATC: Anatomical Therapeutic Chemical
AWaRe: Access Watch Reserve
CT: Consumption Tool
DDD: Defined daily dose
DHIS: Diagnosis and Health Information System
DRC: Democratic Republic of Congo
ICU: Intensive care unit
IDP: Internally displaced person
LMICs: Low- and middle-income countries
MSF: Médecins Sans Frontières
WHO: World Health Organization

## References

[1] Cox JA, Vlieghe E, Mendelson M, Wertheim H, Ndegwa L, Villegas MV, et al. Antibiotic stewardship in low- and middle-income countries: the same but different? Clin Microbiol Infect. 2017;23(11):812–8. 28712667 10.1016/j.cmi.2017.07.010

[2] Nerlich B, James R. The post-antibiotic apocalypse and the war on superbugs: catastrophe discourse in microbiology, its rhetorical form and political function. Public Underst Sci. 2009;18(5):574–90. 20027773 10.1177/0963662507087974

[3] O’Neill J. Review on Antimicrobial Resistance Antimicrobial Resistance: Tackling a crisis for the health and wealth of nations. London; 2014 [cited 2023 Apr 12]. https://amr-review.org/sites/default/files/AMR%20Review%20Paper%20-%20Tackling%20a%20crisis%20for%20the%20health%20and%20wealth%20of%20nations_1.pdf

[4] Bloom G, Merrett GB, Wilkinson A, Lin V, Paulin S. Antimicrobial resistance and universal health coverage. BMJ Glob Health. 2017;2(4):e000518. 29225955 10.1136/bmjgh-2017-000518 PMC5717966

[5] Laxminarayan R, Sridhar D, Blaser M, Wang M, Woolhouse M. Achieving global targets for antimicrobial resistance. Science. 2016;353(6302):874–5. 27540009 10.1126/science.aaf9286

[6] Wernli D, Jørgensen PS, Morel CM, Carroll S, Harbarth S, Levrat N, et al. Mapping global policy discourse on antimicrobial resistance. BMJ Glob Health. 2017;2(2):e000378. 29225939 10.1136/bmjgh-2017-000378 PMC5717922

[7] Kobeissi E, Menassa M, Moussally K, Repetto E, Soboh I, Hajjar M, et al. The socioeconomic burden of antibiotic resistance in conflict-affected settings and refugee hosting countries: a systematic scoping review. Confl Health. 2021;15(1):21. 33823882 10.1186/s13031-021-00357-6 PMC8025481

[8] Pokharel S, Raut S, Adhikari B. Tackling antimicrobial resistance in low-income and middle-income countries. BMJ Glob Health. 2019;4(6):e002104. 31799007 10.1136/bmjgh-2019-002104 PMC6861125

[9] World Health Organization. WHO report on surveillance of antibiotic consumption: 2016–2018 early implementation. Geneva: World Health Organization. 2018 [cited 2023 Apr 14]. 113 p. https://apps.who.int/iris/handle/10665/277359

[10] World Health Organization. Building health system resilience to public health challenges: guidance for implementation in countries. Geneva, Switzerland: WHO. 2024 [cited 2024 Jun 24]. https://www.who.int/publications/i/item/9789240094321

[11] Arepyeva MA, Kolbin AS, Sidorenko SV, Lawson R, Kurylev AA, Balykina YE, et al. A mathematical model for predicting the development of bacterial resistance based on the relationship between the level of antimicrobial resistance and the volume of antibiotic consumption. J Global Antimicrob Resist. 2017;8:148–56. 10.1016/j.jgar.2016.11.010 28167308

[12] Klein EY, Van Boeckel TP, Martinez EM, Pant S, Gandra S, Levin SA et al. Global increase and geographic convergence in antibiotic consumption between 2000 and 2015. Proc Natl Acad Sci USA. 2018 Apr 10 [cited 2023 Jan 21];115(15). 10.1073/pnas.1717295115 10.1073/pnas.1717295115 PMC5899442 29581252

[13] World Health Organization. Global action plan on antimicrobial resistance. Geneva: World Health Organization. 2015 [cited 2023 Apr 10]. 28 p. https://apps.who.int/iris/handle/10665/193736 10.7196/samj.9644 26242647

[14] World Health Organization. More countries committing to tackling antimicrobial resistance. 2021 [cited 2024 Jun 24]. https://www.who.int/news/item/11-11-2021-more-countries-committing-to-tackling-antimicrobial-resistance

[15] World Health Organization. The WHO AWaRe (Access, Watch, Reserve) antibiotic book. Geneva: World Health Organization. 2022 [cited 2023 Jan 9]. https://www.who.int/publications/i/item/9789240062382

[16] World Health Organization. GLASS guide for national surveillance systems for monitoring antimicrobial consumption in hospitals, WHO. 2020 [cited 2023 Apr 5]. https://www.who.int/publications/i/item/9789240000421

[17] Boyles TH, Naicker V, Rawoot N, Raubenheimer PJ, Eick B, Mendelson M. Sustained reduction in antibiotic consumption in a South African public sector hospital; four year outcomes from the Groote Schuur Hospital antibiotic stewardship program. S Afr Med J. 2017;107(2):115. 28220735 10.7196/SAMJ.2017.v107i2.12067

[18] Amaha ND, Weldemariam DG, Berhe YH. Antibiotic consumption study in two hospitals in Asmara from 2014 to 2018 using WHO’s defined daily dose (DDD) methodology. Karunasagar I, editor. PLoS ONE. 2020;15(7):e0233275. 32614832 10.1371/journal.pone.0233275 PMC7332034

[19] Gutema G, Håkonsen H, Engidawork E, Toverud EL. Multiple challenges of antibiotic use in a large hospital in Ethiopia – a ward-specific study showing high rates of hospital-acquired infections and ineffective prophylaxis. BMC Health Serv Res. 2018;18(1):326. 29724214 10.1186/s12913-018-3107-9 PMC5934805

[20] Zay Ya K, Win PTN, Bielicki J, Lambiris M, Fink G. Association between Antimicrobial Stewardship Programs and Antibiotic Use globally: a systematic review and Meta-analysis. JAMA Netw Open. 2023;6(2):e2253806. 36757700 10.1001/jamanetworkopen.2022.53806 PMC9912134

[21] Gebretekle GB, Haile Mariam D, Abebe W, Amogne W, Tenna A, Fenta TG et al. Opportunities and barriers to implementing antibiotic stewardship in low and middle-income countries: Lessons from a mixed-methods study in a tertiary care hospital in Ethiopia. Figueras A, editor. PLoS ONE. 2018;13(12):e0208447. 10.1371/journal.pone.0208447 PMC6301706 30571688

[22] Raut S, Adhikari B. Global leadership against antimicrobial resistance ought to include developing countries. Lancet Infect Dis. 2016;16(7):775. 27352753 10.1016/S1473-3099(16)30078-0

[23] Gayer M, Legros D, Formenty P, Connolly MA. Conflict and emerging infectious diseases. Emerg Infect Dis. 2007;13(11):1625–31. 18217543 10.3201/eid1311.061093 PMC3375795

[24] Haraoui L, Sparrow A, Sullivan R. Armed conflicts and antimicrobial resistance: a deadly convergence. Global Health Secur. 2019;69–73.

[25] Rasheed H, Usman M, Ahmed W, Bacha MH, Zafar A, Bukhari KS. A Shift from Logistic Software to Service Model: a case study of New Service-Driven-Software for Management of Emergency supplies during disasters and Emergency conditions by WHO. Front Pharmacol. 2019;10:473. 31133856 10.3389/fphar.2019.00473 PMC6514185

[26] Kanapathipillai R, Malou N, Hopman J, Bowman C, Yousef N, Michel J, et al. Antibiotic resistance in conflict settings: lessons learned in the Middle East. JAC-Antimicrobial Resist. 2019;1(1):dlz002. 10.1093/jacamr/dlz002 PMC8210113 34222876

[27] Burtscher D, Van Den Bergh R, Nasim M, Mahama G, Au S, Williams A et al. P Shankar editor 2021 ‘They eat it like sweets’: a mixed methods study of antibiotic perceptions and their use among patients, prescribers and pharmacists in a district hospital in Kabul, Afghanistan. PLoS ONE 16 11 e0260096. 34797865 10.1371/journal.pone.0260096 PMC8604360

[28] MSF international. Médecins Sans Frontières. [cited 2023 Jul 13]. https://www.msf.org/

[29] Chukwumeze F, Lenglet A, Olubiyo R, Lawal AM, Oluyide B, Oloruntuyi G, et al. Multi-drug resistance and high mortality associated with community-acquired bloodstream infections in children in conflict-affected northwest Nigeria. Sci Rep. 2021;11(1):20814. 34675262 10.1038/s41598-021-00149-1 PMC8531324

[30] World Health Organization, Collaborating Centre for Drug Statistics Methodology. WHO Collaborating Centre for Drug Statistics Methodology. [cited 2023 Apr 15]. ATC classification index with DDDs. https://www.whocc.no/use_of_atc_ddd/

[31] Bitterman R, Hussein K, Leibovici L, Carmeli Y, Paul M. Systematic review of antibiotic consumption in acute care hospitals. Clin Microbiol Infect. 2016;22(6):e5617–56119. 10.1016/j.cmi.2016.01.026 26899826

[32] Van Boeckel TP, Gandra S, Ashok A, Caudron Q, Grenfell BT, Levin SA, et al. Global antibiotic consumption 2000 to 2010: an analysis of national pharmaceutical sales data. Lancet Infect Dis. 2014;14(8):742–50. 25022435 10.1016/S1473-3099(14)70780-7

[33] World Health Organization. Library of AMR national action plans. [cited 2024 Jun 24]. https://www.who.int/teams/surveillance-prevention-control-AMR/national-action-plan-monitoring-evaluation/library-of-national-action-plans

