# Supplementary material for: Antibiotic consumption in hospitals in humanitarian settings in Afghanistan, Bangladesh, Democratic Republic of Congo, Ethiopia and South Sudan
Source: Antimicrob Resist Infect Control. 2024 Aug 15;13:89. doi: 10.1186/s13756-024-01449-7 (PMC11328513; doi:10.1186/s13756-024-01449-7)
Supplement: Supplementary file 1 — Supplementary Material 1 [file 13756_2024_1449_MOESM1_ESM.docx]

Supplementary Material

**1. Boost hospital**


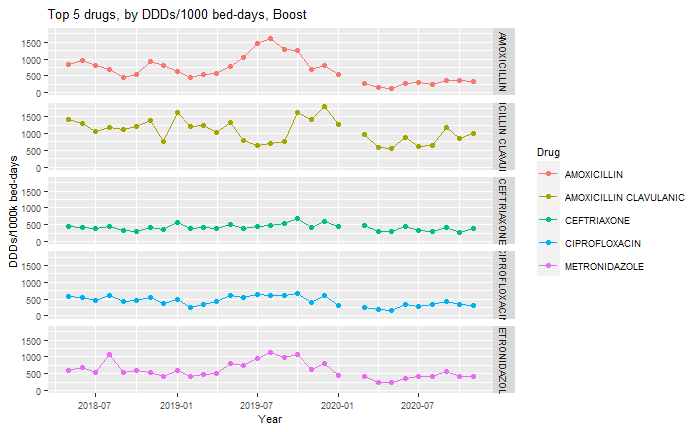


*Figure 3. The most consumed antibiotics by DDDs/1000 bed-days in Boost hospital- Afghanistan between 2018 and 2020.*

**2. Kutupalong hospital**


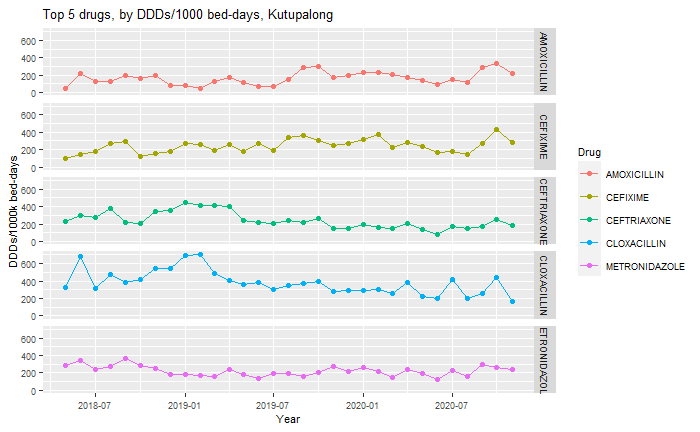


*Figure 4. The most consumed antibiotics by DDDs/1000 bed-days in Kutupalong hospital- Bangladesh between 2018 and 2020.*

**3. Baraka hospital**


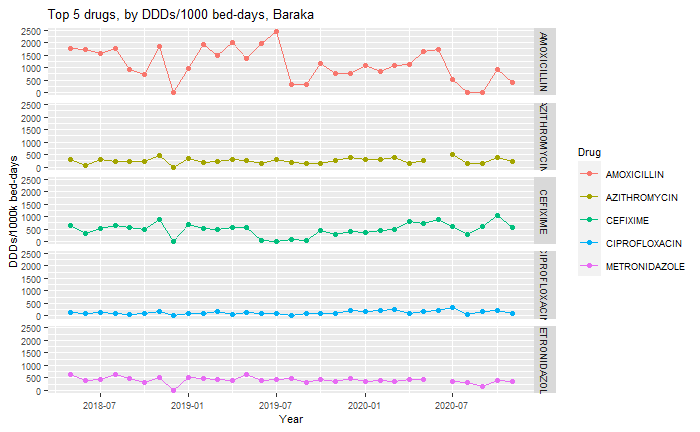


*Figure 5. The most consumed antibiotics by DDDs/1000 bed-days in Baraka hospital- DRC between 2018 and 2020.*

**4. Mweso hospital**


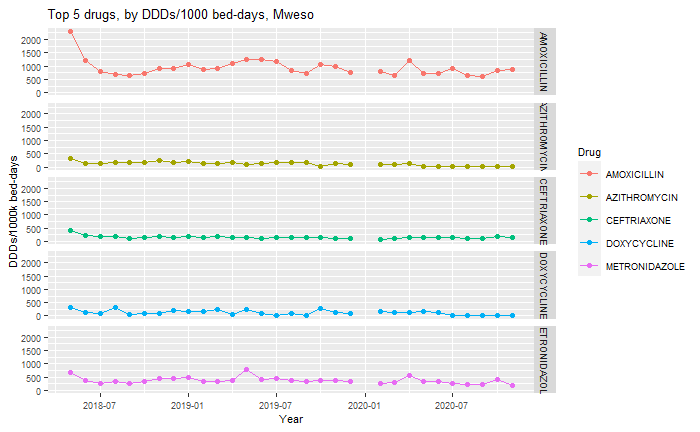


*Figure 6. The most consumed antibiotics by DDDs/1000 bed-days in Mweso hospital- DRC between 2018 and 2020.*

**5. Kule hospital**


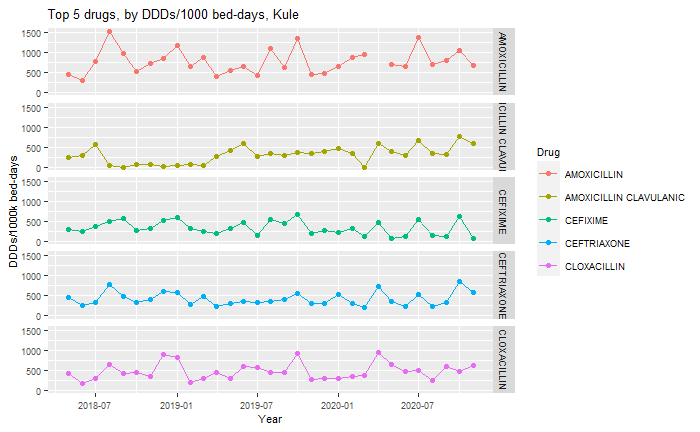


*Figure 7. The most consumed antibiotics by DDDs/1000 bed-days in Kule hospital- Ethiopia between 2018 and 2020.*

**6. Bentiu hospital**


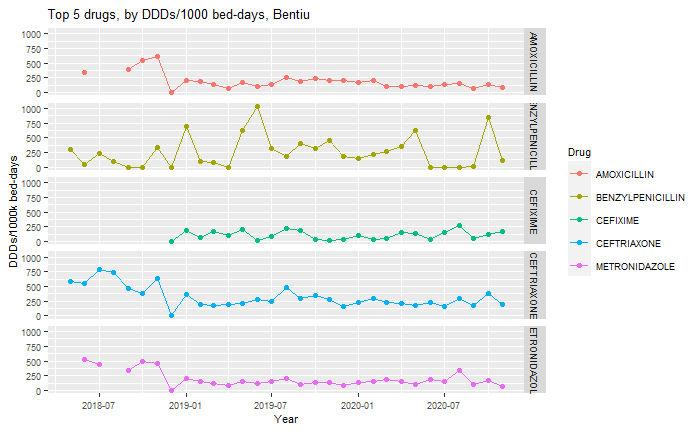


*Figure 8. The most consumed antibiotics by DDDs/1000 bed-days in Bentiu hospital- South Sudan between 2018 and 2020.*
